# Genome-wide associations for immune traits in two maternal pig lines

**DOI:** 10.1186/s12864-021-07997-1

**Published:** 2021-10-05

**Authors:** Christina M. Dauben, Maren J. Pröll-Cornelissen, Esther M. Heuß, Anne K. Appel, Hubert Henne, Katharina Roth, Karl Schellander, Ernst Tholen, Christine Große-Brinkhaus

**Affiliations:** 1 Institute of Animal Sciences, University of Bonn, Endenicher Allee 15, Bonn, 53115 Germany; 2 BHZP GmbH, An der Wassermühle 8, Dahlenburg-Ellringen, 21368 Germany

**Keywords:** Immune system, Pigs, Genome-wide Association Studies, Immunocompetence, Animal Genetics

## Abstract

**Background:**

In recent years, animal welfare and health has become more and more important in pig breeding. So far, numerous parameters have been considered as important biomarkers, especially in the immune reaction and inflammation. Previous studies have shown moderate to high heritabilities in most of these traits. However, the genetic background of health and robustness of pigs needs to be extensively clarified. The objective of this study was to identify genomic regions with a biological relevance for the immunocompetence of piglets. Genome-wide Association Studies (GWAS) in 535 Landrace (LR) and 461 Large White (LW) piglets were performed, investigating 20 immune relevant traits. Besides the health indicators of the complete and differential blood count, eight different cytokines and haptoglobin were recorded in all piglets and their biological dams to capture mediating processes and acute phase reactions. Additionally, all animals were genotyped using the Illumina PorcineSNP60v2 BeadChip.

**Results:**

In summary, GWAS detected 25 genome-wide and 452 chromosome-wide significant SNPs associated with 17 immune relevant traits in the two maternal pig lines LR and LW. Only small differences were observed considering the maternal immune records as covariate within the statistical model. Furthermore, the study identified across- and within-breed differences as well as relevant candidate genes. In LR more significant associations and related candidate genes were detected, compared with LW. The results detected in LR and LW are partly in accordance with previously identified quantitative trait loci (QTL) regions. In addition, promising novel genomic regions were identified which might be of interest for further detailed analysis. Especially putative pleiotropic regions on SSC5, SSC12, SSC15, SSC16 and SSC17 are of major interest with regard to the interacting structure of the immune system. The comparison with already identified QTL gives indications on interactions with traits affecting piglet survival and also production traits.

**Conclusion:**

In conclusion, results suggest a polygenic and breed-specific background of immune relevant traits. The current study provides knowledge about regions with biological relevance for health and immune traits. Identified markers and putative pleiotropic regions provide first indications in the context of balancing a breeding-based modification of the porcine immune system.

**Supplementary Information:**

The online version contains supplementary material available at (10.1186/s12864-021-07997-1).

## Background

Health and immune traits are characterising the immunocompetence of piglets [1–3]. Through the last years, the societal importance of health and animal welfare has increased [4]. The enhancement of immunocompetence is a potential determinant to prevent performance reduction through health impairment [5, 6]. In literature, several approaches to influence and improve the porcine immune response and immune system have been discussed (e.g., [2, 7, 8]). In this context, haematological parameters and cytokines might be considered as important markers of immune reaction [9, 10]. As the immune system is known as a highly interactive system [11], indicators triggering a health-promoting immunocompetence need to be investigated regarding negative impacts and interactions on economical and societal phenotypes [3, 5].

Piglets are extremely susceptible to infections in their first stage of life [12]. For this reason, innate immune defense mechanisms and colostrum intake are essential to newborn’s immunity [13]. Consequently, it is suggested that the immunocompetence of the biological dam is linked to the immune status of the piglet due to several components of colostrum [13–15].

### Genetic background

Previous studies have estimated heritabilities under various conditions regarding e.g., breed, age (including developmental stage), number of animals and the experimental design (e.g., [1, 3, 5, 10, 16–20]). These studies have shown mostly moderate to high heritabilities for several components of the immune system. Overall, the innate and adaptive immune system indicate genetic foundation and slightly higher individual genetic variances in traits of the adaptive immune system [3, 21]. Especially immune cells, red blood cell (RBC) characteristics and cytokines suggest a large genetic component [3, 19]. Until now, a small number of studies investigated genetic parameters for cytokines in pigs (e.g., [3, 18]).

Heritability estimation in a Landrace and Large White population under non-challenging conditions has confirmed findings to a large extent [22, 23]. RBC and their characteristics showed heritabilities between 0.3 and 0.8 in Landrace and between 0.5 and 0.6 in Large White. In cytokines, heritability estimates were in a broad, breed-specific range from almost 0 to high values of 0.7 (Tumor necrosis factor (TNF)- *α*) in LR and 0.4 (Interleukin (IL)-8) in LW. Therefore, genetic foundation of immune relevant traits and responsiveness for selective breeding is assumed.

Although the genetic background of the porcine immune system still remains unclear, there are some studies trying to identify immune relevant quantitative trait loci (QTL). So far, the number of QTL associated with health and immune response traits in pigs increased from a limited number in 2007 [24] to a moderate number in 2020. According to the latest release of the AnimalQTLdb (Release 43, December 2020, https://www.animalgenome.org/cgi-bin/QTLdb/SS/summary) [25], 2,776 QTL representing associations with blood parameters, 3,231 QTL with immune capacity, and 609 QTL with disease susceptibility were published.

Underlying studies focused on classical haematological parameters, characterised by measurements of the blood count. First QTL were detected using microsatellites (e.g., [26, 27]) or concentrated on particular genes (e.g., [28–30]). Other studies to detect immune relevant QTL vary widely in treatment, methods of phenotype determination and breed (e.g., [10, 31–37]). In addition, different ages and developmental stages are covered, limiting the comparability of the studies. Only a limited number of studies identified QTL associated with the acute phase protein haptoglobin [38] or cytokines. Studies investigating the cytokines Interferon (IFN)- *γ* and IL-10 used vaccination with *Mycoplasma hyopneumoniae*, tetanus toxoid, the porcine reproductive and respiratory syndrome virus (PRRSV) [39, 40] and the classical swine fever virus [41, 42]. Another study analysing cytokines, including IL-6 and IL-12, was restricted to exonic regions of a single gene [43].

### Aim of the study

The aim of this study was to identify biological relevant markers associated with health and immune traits in piglets under non-challenge conditions in a Landrace (LR) and a Large White (LW) population. Besides, the genetic background of the piglets’ immune system and the role of the maternal immunocompetence is discussed.

## Results

In total, 535 piglets of LR and 461 piglets of LW were phenotyped for the complete and differential blood count (15 traits), eight cytokines and haptoglobin in an experiment conducted under mostly practical, but high hygienic conditions and without challenging the animals. The number of samples per trait, least-squares means and standard errors are listed in Table 1. Slight differences in the number of records resulted from laboratory techniques. Differences between the breeds were tested using a generalized linear mixed model corrected for the environmental effects of breed, age and weight at sample collection and their interaction, a combined herd, year, season, sex effect and litter number (model 0). Due to the analytical method, batch was an additional fixed effect and biological dam an additional random effect in the analyses of cytokines. In general, there were significant differences in blood measurements between the breeds, especially in red blood cell characteristics and cytokines. Further differences were identified in the percentages of neutrophils and lymphocytes. The ratio of these closely antagonistic linked traits was above 1 (1.06) in LR but noticeable below 1 (0.76) in the LW population.

**Table 1:** Sample number, least-squares mean (LS-Mean) and standard error of the mean (SEM) for immune relevant traits in piglets of Landrace (LR) and Large White (LW)

		LR			LW			
Trait		n	LS-Mean	SEM	n	LS-Mean	SEM	Breed
WBC	[G/l]	502	20.80	0.46	420	19.29	0.35	*
Neutrophils	[%]	502	50.54	0.84	420	40.35	0.65	***
Lymphocytes	[%]	502	43.19	0.84	420	53.77	0.65	***
Monocytes	[%]	502	3.53	0.14	420	3.76	0.11	
Eosinophils	[%]	502	2.56	0.12	420	1.92	0.09	***
Basophils	[%]	502	0.08	0.03	420	0.09	0.02	
Band cells	[%]	502	0.01	0.01	420	0.02	0.01	
Other cells	[%]	502	0.02	0.01	420	0.01	0.01	
Platelets	[G/l]	502	325.63	12.23	420	358.98	9.42	
Haptoglobin	[mg/ml]	531	0.51	0.03	458	0.49	0.02	
RBC	[T/l]	502	6.37	0.05	420	5.86	0.04	***
Haemoglobin	[g/l]	502	119.74	0.99	420	104.70	0.76	***
Haematocrit	[l/l]	502	0.39	0.00	420	0.34	0.00	***
MCV	[fl]	502	61.66	0.27	420	58.40	0.21	***
MCH	[pg]	502	18.76	0.09	420	17.94	0.07	***
MCHC	[g/dl]	502	30.47	0.11	420	30.73	0.08	
IFN- *γ*	[ng/mL]	501	1.58	0.26	442	1.06	0.15	**
IL-10	[ng/mL]	512	0.50	0.06	447	0.31	0.03	**
IL-12	[ng/mL]	512	0.56	0.02	447	0.69	0.02	
IL-1 *β*	[ng/mL]	512	0.45	0.05	447	0.32	0.03	**
IL-4	[ng/mL]	512	0.50	0.08	447	0.26	0.04	**
IL-6	[ng/mL]	512	0.16	0.02	447	0.08	0.01	**
IL-8	[ng/mL]	512	0.48	0.04	447	0.38	0.02	
TNF- *α*	[ng/mL]	512	0.07	0.01	447	0.05	0.00	***

*Breed* Significance of breed differences, *WBC* White blood cells, *RBC* Red blood cells, *MCV* Mean corpuscular volume, *MCH* Mean corpuscular haemoglobin, *MCHC* Mean corpuscular haemoglobin concentration, *IFN* Interferon, *IL* Interleukin, *TNF* Tumor necrosis factor, *p-values* 0.05 * 0.01 ** 0.001 *** 0

### Genome-wide association studies

**Fig. 1:**
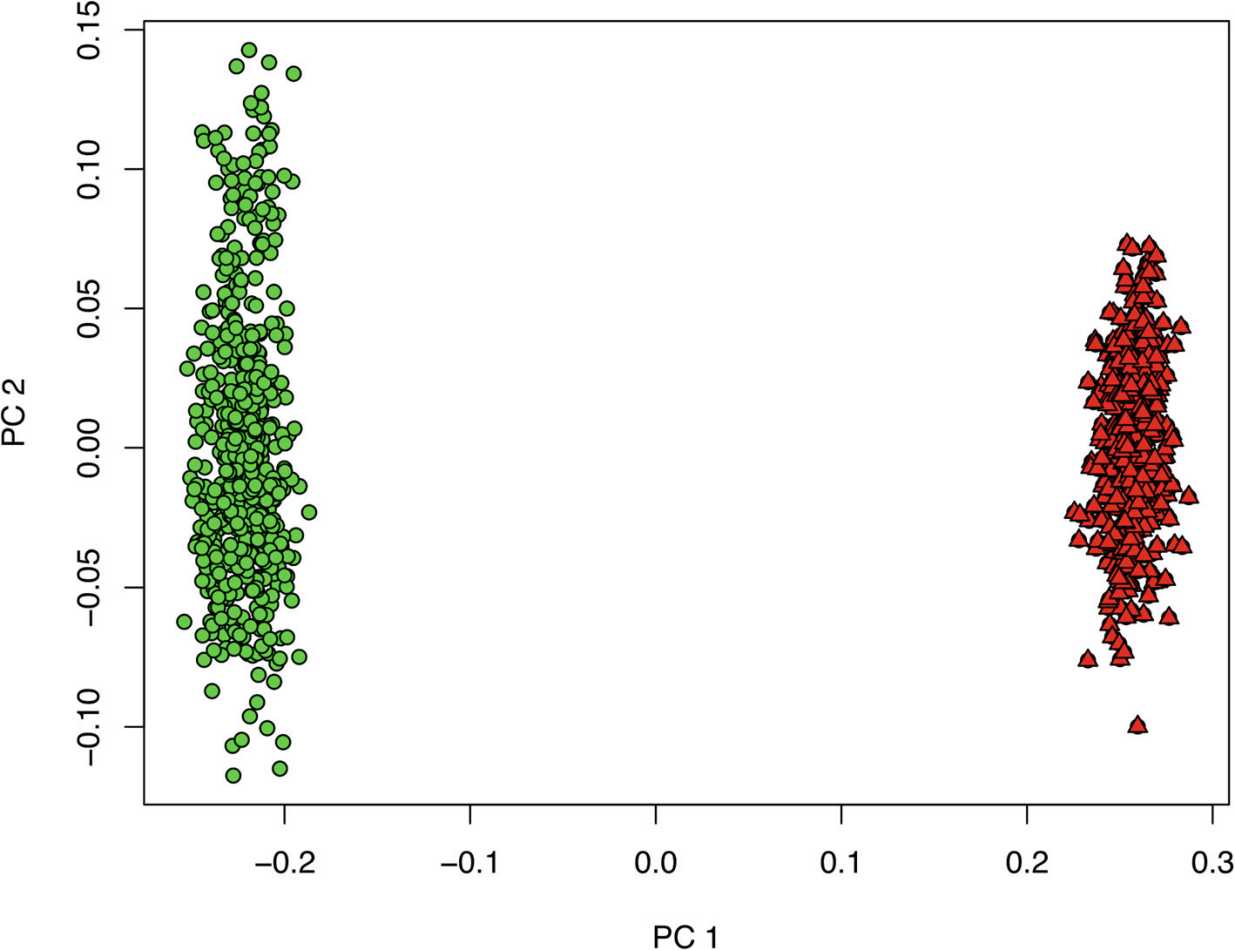
Genetic distances between the analysed animals from Landrace (green circle) and Large White (red triangle) displayed as the first two principal components (PC)

Different genetic backgrounds led to breed-specific investigations of LR and LW (see Fig. 1). For identifying associations between SNP genotypes and phenotypic information, model (0) without the fixed breed effect was extended by a fixed SNP effect and further on denoted as model (1). Genome-wide association studies (GWAS) based on model (1) identified a total of 25 genome-wide and 452 chromosome-wide significant SNPs (p ≤0.05, corrected for q-value) associated with 17 traits characterising the immunocompetence in unchallenged pigs (Table 2). As expected from more conservative correction methods to avoid false positive results, applying the Bonferroni threshold of 0.05 confirmed 12.8% (LR) and 10.7% (LW) of those markers. With the Bonferroni threshold for adjusted *p*-values of 0.1, the amount of validated SNPs was increased up to 17.9% (LR) and 13.2% (LW).

**Table 2:** Number of significant SNPs on a chromosome-wide (genome-wide) level

Trait	Landrace	Large White
White blood cells (WBC)	80	0
Neutrophils	35	0
Lymphocytes	0	0
Monocytes	4	6
Platelets	0	8
Haptoglobin	7	2
Red blood cells (RBC)	0	18
Haemoglobin	0	4
Haematocrit	7	2
Mean corpuscular volume (MCV)	0	0
Mean corpuscular haemoglobin (MCH)	0	0
Mean corpuscular haemoglobin concentration (MCHC)	28	14
Interferon (IFN)- *γ*	0	1
Interleukin (IL)-10	11	1
IL-12	0	130
IL-1 *β*	7	0
IL-4	10	0
IL-6	0	2
IL-8	107 (23)	0
Tumor necrosis factor (TNF)- *α*	0	9 (2)
Total	280 ^*#*^	197

^#^SNPs with a significance in multiple traits are counted once

Most of the associations are variants within intergenic (LR: 119, LW: 94) or intronic (LR: 142, LW: 89) regions. A lower number of significant SNPs is located within untranslated (3-prime-UTR variant, 5-prime-UTR variant) or upstream and downstream regions of a gene. In addition, GWAS on immune relevant traits identified a few synonymous variants and a variant within a splice site (IL-12, LW) or a non-coding exon sequence (WBC, LR) as well as a missense variant (haemoglobin, LW). Each significant SNP explained between 1.7-5.2% and 2.2-5.5% of the phenotypic variance in LR and LW, respectively.

Tables 3 and 4 provide an overview of the Top 5 SNPs for each immune relevant trait analysed, explaining the largest proportion of the phenotypic variance. A complete list of significant SNPs is given in Additional files 1 and 2.

**Table 3:** Top 5 SNPs explaining the largest phenotypic variance for immune relevant traits identified by GWAS in Landrace

Trait	SNP	SSC	Pos.	Var.	MAF	*p*-value		rs code	SNP type	Gene
WBC	H3GA0014703	4	124.4	4.5	0.317	2.53E-05	B10	rs80835478	IV	EVI5
WBC	H3GA0014699	4	124.5	4.3	0.320	3.44E-05	B10	rs80930480	IV	EVI5
WBC	MARC0027349	12	21.7	4.3	0.150	3.48E-05	B5	rs81222578	IV	ENSSSCG00000043259
WBC	ALGA0077342	14	45.0	4.0	0.195	6.79E-05		rs80964268	IGV	
WBC	ISU10000541	14	47.2	4.1	0.282	5.64E-05		rs80863047	3pUTR	LIF
NEU	ALGA0121271	12	19.4	4.0	0.355	1.65E-04		rs81328838	UGV	MEOX1
NEU	ALGA0113322	12	27.6	3.8	0.421	2.16E-04		rs81342064	IGV	
NEU	ALGA0066053	12	33.0	3.8	0.377	2.45E-04		rs81433794	IGV	
NEU	DRGA0011723	12	33.1	3.8	0.377	2.45E-04		rs81294298	IV	C17orf67
NEU	ASGA0102924	12	60.3	4.0	0.386	1.41E-04		rs81324324	IV	FAM83G, SLC5A10
MON	H3GA0034268	12	36.0	4.1	0.159	4.18E-05	B10	rs81434487	IV	VMP1
MON	ALGA0066234	12	36.0	3.9	0.162	5.33E-05	B10	rs81434489	IV	VMP1
MON	ASGA0054380	12	36.1	3.9	0.162	5.33E-05	B10	rs81434493	IV	TUBD1
MON	ALGA0067033	12	54.0	3.7	0.077	7.93E-05		rs81437196	IV	PIK3R5
HAP	ALGA0013060	2	37.2	4.5	0.061	8.70E-06	B5	rs81357336	IV	ANO5
HAP	DRGA0002935	2	37.2	4.5	0.061	8.72E-06	B5	rs81296277	IV	ANO5
HAP	ALGA0013078	2	37.8	4.5	0.061	8.70E-06	B5	rs81357366	IGV	
HAP	ALGA0053703	9	69.8	4.7	0.470	6.25E-06	B5	rs81413020	IGV	
HAP	ALGA0053704	9	69.9	5.0	0.450	2.84E-06	B5	rs81413026	IGV	
HCT	ASGA0002880	1	53.5	5.2	0.261	2.41E-05		rs80853363	IGV	
HCT	M1GA0000961	1	53.9	4.5	0.227	8.90E-05		rs80858850	UGV	TBX18
HCT	INRA0002424	1	54.0	5.1	0.266	3.28E-05		rs341132099	IGV	
HCT	INRA0002425	1	54.0	4.5	0.227	8.90E-05		rs332231926	IGV	
HCT	ALGA0003516	1	54.1	5.1	0.266	3.28E-05		rs80921572	IGV	
MCHC	H3GA0016097	5	23.4	3.1	0.449	6.16E-05		rs80964787	IGV	
MCHC	ALGA0032074	5	58.6	3.0	0.015	9.13E-05		rs80787531	IV	GRIN2B
MCHC	H3GA0016359	5	58.6	3.0	0.015	9.13E-05		rs80942872	IV	GRIN2B
MCHC	H3GA0016379	5	58.8	3.0	0.015	9.13E-05		rs80933182	IV	GRIN2B
MCHC	ALGA0032146	5	59.3	3.0	0.015	9.13E-05		rs81384359	IGV	
IL-10	ALGA0094933	17	39.0	3.7	0.122	1.95E-05	B5	rs80997686	UGV	NFS1, ROMO1, RBM39
IL-10	ALGA0094942	17	39.2	3.7	0.122	1.95E-05	B5	rs81466504	IGV	
IL-10	ASGA0076872	17	39.2	3.7	0.122	1.95E-05	B5	rs81466495	IGV	
IL-10	ASGA0076896	17	39.4	3.7	0.122	1.95E-05	B5	rs80827927	IV	EPB41L1
IL-10	ASGA0076900	17	39.5	3.7	0.122	1.95E-05	B5	rs80842622	IV	EPB41L1
IL-1 *β*	ALGA0094933	17	39.0	2.9	0.122	1.63E-04		rs80997686	UGV	NFS1, ROMO1, RBM39
IL-1 *β*	ALGA0094942	17	39.2	2.9	0.122	1.63E-04		rs81466504	IGV	
IL-1 *β*	ASGA0076872	17	39.2	2.9	0.122	1.63E-04		rs81466495	IGV	
IL-1 *β*	ASGA0076896	17	39.4	2.9	0.122	1.63E-04		rs80827927	IV	EPB41L1
IL-1 *β*	ASGA0076900	17	39.5	2.9	0.122	1.63E-04		rs80842622	IV	EPB41L1
IL-4	ALGA0039474	7	22.1	4.0	0.303	6.04E-06	B5	rs81002011	IV	ZSCAN26, PGBD1
IL-4	ALGA0094933	17	39.0	2.8	0.122	1.61E-04		rs80997686	UGV	NFS1, ROMO1, RBM39
IL-4	ALGA0094942	17	39.2	2.8	0.122	1.61E-04		rs81466504	IGV	
IL-4	ASGA0076872	17	39.2	2.8	0.122	1.61E-04		rs81466495	IGV	
IL-4	ASGA0076896	17	39.4	2.8	0.122	1.61E-04		rs80827927	IV	EPB41L1
IL-8	ALGA0086892	15	116.1	5.1	0.494	2.86E-07 *	B5*	rs81454413	IV	SPAG16
IL-8	ASGA0070560	15	120.0	4.6	0.256	1.11E-06 *	B5*	rs81454672	IV	TNS1
IL-8	ASGA0070582	15	120.1	4.7	0.257	8.45E-07 *	B5*	rs81454730	IV	TNS1
IL-8	ASGA0070586	15	120.1	5.1	0.417	2.87E-07 *	B5*	rs80818610	IV	TNS1
IL-8	ALGA0087116	15	120.3	5.0	0.428	4.19E-07 *	B5*	rs80913177	IGV	

*WBC* White blood cells, *NEU* Neutrophils, *MON* Monocytes, *HAP* Haptoglobin, *HCT* Haematocrit, *MCHC* Mean corpuscular haemoglobin concentration, *IL* Interleukin, *SSC* Sus Scrofa Chromosome, *Pos.* Position [Mb], *Var.* Phenotypic variance explained [%], *MAF* Minor allele frequency, * genome-wide significance, *B5* Additionally significant after Bonferroni correction with adjusted *p*-value <0.05, *B10* Additionally significant after Bonferroni correction with adjusted *p*-value <0.1, *Gene* SNP within gene, *IV* Intron variant, *IGV* Intergenic variant, *3pUTR* 3 prime UTR variant, *UGV* Upstream gene variant

**Table 4:** Top 5 SNPs explaining the largest phenotypic variance for immune relevant traits identified by GWAS in Large White

Trait	SNP	SSC	Pos.	Var.	MAF	*p*-value		rs code	SNP type	Gene
MON	H3GA0050761	18	37.3	4.3	0.458	1.41E-04		rs81469020	IGV	
MON	H3GA0050788	18	38.3	4.1	0.466	2.05E-04		rs81469187	IGV	
MON	ALGA0098112	18	39.3	4.0	0.462	2.51E-04		rs81469256	IGV	
MON	MARC0069672	18	39.3	4.0	0.463	2.38E-04		rs81255938	IGV	
MON	MARC0089391	18	39.3	4.0	0.462	2.51E-04		rs81270496	IGV	
PLT	ASGA0029231	6	113.4	4.1	0.026	1.70E-05	B5	rs81390902	IGV	
PLT	ASGA0029288	6	120.2	4.1	0.027	1.71E-05	B5	rs81391141	IV	FHOD3
PLT	ALGA0057477	10	17.2	3.6	0.383	5.42E-05	B10	rs81421220	IGV	
PLT	H3GA0053711	10	17.4	4.0	0.377	2.19E-05	B5	rs81345552	IGV	
PLT	ALGA0116316	10	17.5	3.4	0.331	9.22E-05		rs81345791	IV	HNRNPU
HAP	ALGA0022724	4	7.5	4.1	0.300	1.27E-05	B5	rs80997926	IGV	
HAP	ALGA0096031	17	54.9	3.7	0.298	3.18E-05	B5	rs80828451	IV	BCAS1
RBC	ALGA0066876	12	50.1	5.0	0.318	2.70E-05	B5	rs81436461	IV	ZZEF1
RBC	ALGA0066881	12	50.1	5.0	0.318	2.70E-05	B5	rs81436486	IV	ZZEF1
RBC	ALGA0079331	14	83.0	4.8	0.065	4.43E-05		rs80806469	IGV	
RBC	SIRI0000773	14	136.4	5.3	0.084	1.69E-05		rs325538072	IV	INSYN2A, DOCK1
RBC	ASGA0074790	16	78.0	5.5	0.011	1.21E-05	B5	rs81463953	IGV	
HGB	ASGA0025952	5	65.8	5.4	0.203	6.80E-06	B5	rs81384737	MV	AKAP3
HGB	H3GA0016570	5	66.0	5.3	0.202	9.73E-06	B5	rs80994174	IV	FGF6
HGB	ALGA0085557	15	56.0	5.4	0.014	7.79E-06	B5	rs81453155	IGV	
HGB	ASGA0074790	16	78.0	4.7	0.011	2.96E-05	B5	rs81463953	IGV	
HCT	ALGA0085557	15	56.0	5.0	0.014	5.43E-06	B5	rs81453155	IGV	
HCT	ASGA0074790	16	78.0	4.9	0.011	5.90E-06	B5	rs81463953	IGV	
MCHC	ASGA0005789	1	221.2	4.9	0.126	6.61E-05		rs80903521	IV	KANK1
MCHC	MARC0079029	1	254.2	4.8	0.181	7.18E-05		rs81263277	IV	RGS3
MCHC	ASGA0102333	1	254.2	4.9	0.182	6.95E-05		rs81323628	IGV	
MCHC	H3GA0053907	1	254.2	4.8	0.181	7.18E-05		rs81347166	IGV	
MCHC	MARC0026691	1	255.2	4.9	0.148	6.05E-05		rs80929320	IV	ATP6V1G1
IFN- *γ*	H3GA0011038	3	128.4	4.3	0.318	1.49E-05	B5	rs81378478	IGV	
IL-10	MARC0016481	13	17.5	4.8	0.221	8.34E-06	B5	rs81285895	IGV	
IL-12	INRA0000110	1	3.1	3.8	0.312	6.29E-05		rs80934703	IV	PDE10A
IL-12	MARC0102958	1	3.2	3.8	0.312	6.29E-05		rs80961411	IV	PDE10A
IL-12	ALGA0000305	1	3.3	3.8	0.312	6.29E-05		rs80793535	IV	PDE10A
IL-12	DRGA0012960	13	152.9	3.8	0.499	5.77E-05		rs81298281	IV	ENSSSCG00000042450
IL-12	ASGA0092143	13	153.6	4.0	0.500	3.33E-05		rs81478305	IV	CBLB
IL-6	ASGA0051648	11	68.5	4.3	0.055	2.39E-05	B5	rs81431737	IV	CLYBL
IL-6	H3GA0032382	11	68.6	3.9	0.042	5.45E-05	B10	rs80961677	IV	CLYBL
TNF- *α*	ASGA0001772	1	26.5	3.8	0.446	1.78E-05	B10	rs81351651	IGV	
TNF- *α*	ASGA0001781	1	26.7	4.9	0.498	8.52E-07 *	B5*	rs80894799	IV	ENSSSCG00000043500
TNF- *α*	ASGA0105343	9	138.4	3.9	0.159	1.26E-05	B5	rs81305425	IGV	
TNF- *α*	ASGA0097568	9	138.5	4.9	0.064	7.99E-07 *	B5*	rs81317558	IGV	
TNF- *α*	ALGA0056053	9	138.8	4.2	0.062	5.21E-06	B5	rs81419664	IGV	

*MON* Monocytes, *PLT* Platelets, *HAP* Haptoglobin, *RBC* Red blood cells, *HGB* Haemoglobin, *HCT* Haematocrit, *MCHC* Mean corpuscular haemoglobin concentration, *IFN* Interferon, *IL* Interleukin, *TNF* Tumor necrosis factor, *SSC* Sus Scrofa Chromosome, *Pos.* Position [Mb], *Var.* phenotypic variance explained [%], *MAF* Minor allele frequency, * genome-wide significance, *B5* Additionally significant after Bonferroni correction with adjusted *p*-value <0.05, *B10* Additionally significant after Bonferroni correction with adjusted *p*-value <0.1, *Gene* SNP within gene, *IGV* Intergenic variant, *IV* Intron variant, *MV* Missense variant

Due to the unclear influence of the dam on the immunocompetence of the piglets, model (1) was extended by the corresponding trait measured in the biological dam, included as a fixed covariate (model 1^*^). The effect was significant in twelve (LR) and ten (LW) traits out of 20. GWAS using model (1^*^) confirmed the majority (LR: 206, LW: 127) of the significant genomic regions from basic analysis using model (1). In some traits without any significant SNP (TNF- *α* in LR, lymphocytes and IL-4 in LW), model (1^*^) revealed single markers which were among the leading SNPs identified by model (1), but did not exceed the chromosome-wide threshold. Consequently, the benefit of model (1*) is limited. Conspicuous deviations from results based on model (1) were identified in IL-6 in LW. Markers associated with IL-6 diverged in the chromosomal region.

Due to the overall minor role of the additional covariate, only results of model (1) are discussed further.

Regarding the genomic background within the breeds, remarkably more significances were found within LR (n _*SNPs*_ = 296) compared to LW (n _*SNPs*_ = 197). Significant genomic regions of different traits did not overlap in a window of 70 kb. Window definition followed the twofold mean distance of markers identified by Ramos et al. [44].

With exception of lymphocytes, mean corpuscular volume (MCV) and mean corpuscular haemoglobin (MCH), significant SNPs were identified for all traits analysed. In most cases, SNPs were only found within the LR or LW. For five traits (monocytes, haptoglobin, haematocrit, mean corpuscular haemoglobin concentration (MCHC), IL-10), detected markers were relevant in both breeds, but not in overlapping chromosomal regions. Moreover, WBC, neutrophils, IL-1 *β*, IL-4, and IL-8 were found to be only associated within LR. In contrast, haematopoietic traits, such as platelets, RBC, and haemoglobin as well as the cytokines IFN- *γ*, IL-12, IL-6 and TNF- *α* only showed associated SNPs within LW.

Identified SNPs were located across all autosomes with the exception of SSC8. But there were chromosome-specific clusters for certain trait complexes. For example, SSC12 showed associations with cell parameters such as WBC, neutrophils, monocytes, and RBC, whereas on SSC16 only associations with traits characterising RBC in LW were observed.

The largest number of significant markers was found for cytokines (Table 2) with an important region located on SSC17 in LR. In a narrow region between 37 and 39 to 40 Mb on SSC17, significantly associated SNPs for IL-1 *β*, IL-10 and IL-4 in LR were identified. These SNPs explained up to 3.7% of the phenotypic variance and associations with IL-10 were additionally confirmed with the more conservative Bonferroni correction at 5%.

In addition, 23 SNPs for IL-8 in LR and two SNPs for TNF- *α* in LW exceeded the genome-wide significance level according to q-value and partly the Bonferroni correction and explained up to 5.1% of the phenotypic variance.

Blood cell characteristics accounted for the second largest proportion of chromosome-wide significant SNPs in LR. Monocyte percentage, as the only subset of WBC showing significant SNPs in LR and LW, revealed SNPs on SSC12 and SSC18, respectively.

Putative pleiotropic SNPs were detected on SSC15 (*ALGA0085557*, 56.0 Mb) and SSC16 (*ASGA0074790*, 78.0 Mb) in LW as well as a genomic region on SSC17 in LR, affecting several cytokines.

**Fig. 2:**
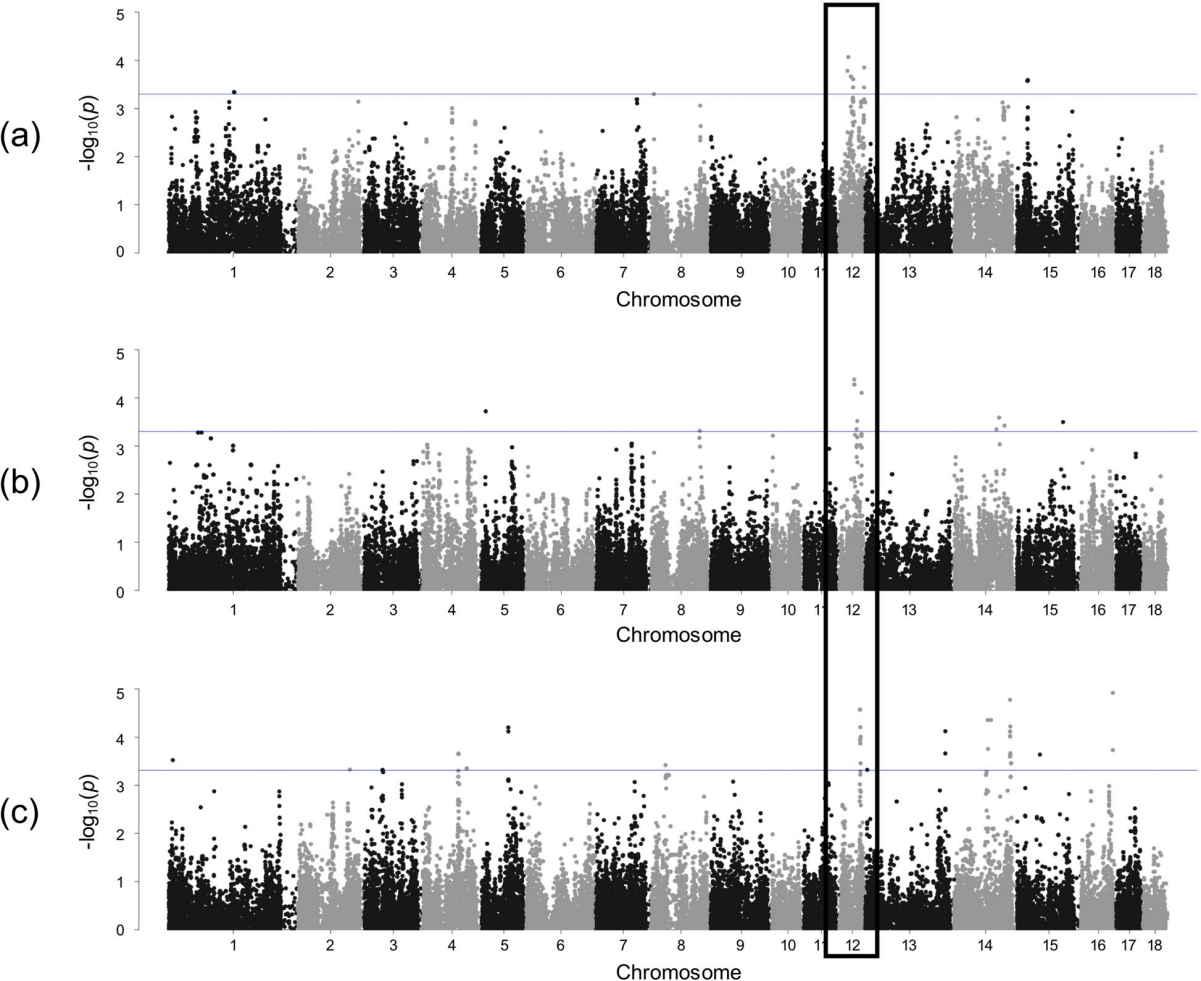
Manhattan plots focusing on a putative pleiotropic region on SSC12 showing results in **a** neutrophils in Landrace, **b** monocytes in Landrace and **c** red blood cells in Large White

**Fig. 3:**
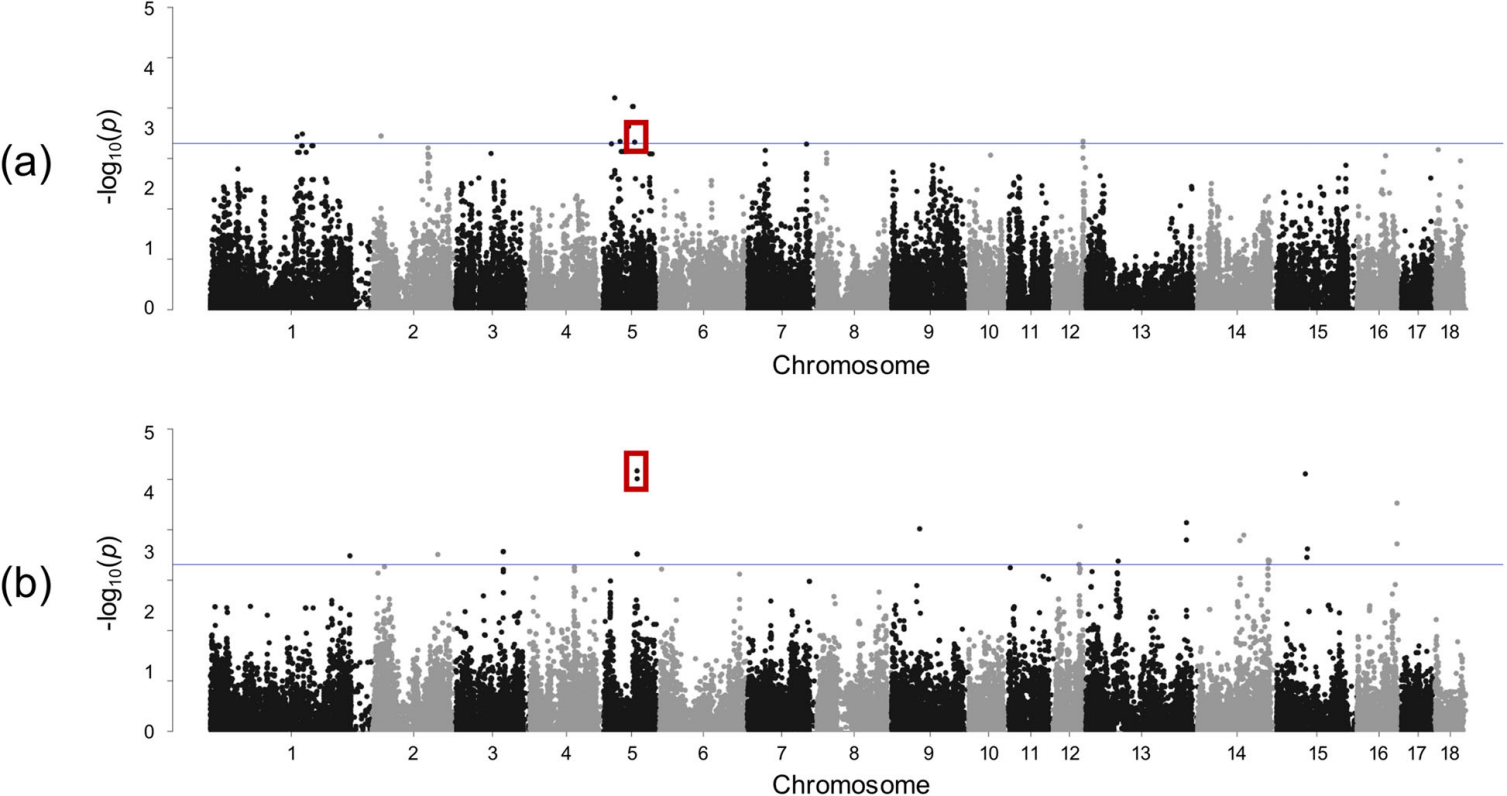
Manhattan plots focusing on a putative pleiotropic region on SSC5 showing results in **a** MCHC in Landrace and **b** haemoglobin in Large White

As has been shown, there were no overlapping significant SNPs for traits within LR and LW breed. However, SSC12 seems to be important for both breeds (see Fig. 2). Within a region of around 3.9 Mb, 16 SNPs were found significantly associated with nonidentical immune relevant traits in LR (neutrophils, monocytes) and LW (RBC). A similar observation was made on SSC5 (see Fig. 3). Within an interval of approximately the same size, a significant genomic region was found for MCHC in LR and haemoglobin in LW. In addition, several of the putative pleiotropic SNPs indicated significance under the more conservative Bonferroni correction.

## Discussion

Animal welfare and health are of social and ethical concerns in pig production and science [4]. In recent years, heritability of immune relevant traits has been confirmed (e.g., [1, 3, 5, 10, 16–20, 22, 23]). In this regard, the interacting and networking structure of immune traits, and basically the genetic background of health and robustness of animals needs to be refined (e.g., [6, 20, 36]). Therefore, this study identified biological relevant SNPs associated with traits characterising the immunocompetence of piglets.

Blood counts recorded in this study reflect the situation under high hygienic conditions in nucleus herds. Results indicate first insights into the genetic background of a LR and LW population in a non-challenging experimental design. Hence, these results may hardly be interpreted as reference values for pigs in commercial fattening systems, but they can be used as indicators for the genetic variation of the immune system.

In order to evaluate and characterise the immune status, reference values are needed. Such thresholds were determined by, for example, Seutter [45]. However, due to accelerated genetic progress accompanied by substantial changes in breeding objectives in the recent years, an update of physiological reference values is indispensable.

At the time point of blood sampling, mean concentrations in the here studied LR and LW populations differed, suggesting a diverging ability for immunocompetence at the critical time point around weaning. LW showed a clear lymphocytic blood count (N:L ratio = 0.76), whereas LR tended towards a granulocytic blood count (N:L ratio = 1.06). This led to the assumption of a more specific and higher responding immunocompetence of LW in the first weeks of age. On the other side, the neutrophil-to-lymphocyte ratio is influenced by non-genetic factors like stress [46] or age at blood sampling. However, taking into account that piglets of both breeds were raised under the same high hygienic and husbandry conditions, substantially deviating stress conditions were unlikely.

The impact of age at blood sampling has been discussed in contrasting ways. For one thing, neutrophils have been described as age-independent at an age of around seven weeks [16], whereas another study showed an effect in piglets up to an age of twelve weeks [45]. In our study, blood samples were collected from LR and LW piglets at an average age of 42 (SD = 3.3) to 44 (SD = 5.8) days. Estimated regression coefficients for age in neutrophils were different in LR (0.018% per day) and LW (0.669% per day). Minor importance of age in LR and higher importance in LW conflict with the conclusions of Seutter [45] and Henryon et al. [16] mentioned above. Contradictions might be due to genetic changes in modern breeds within the last decades.

### Genome-wide association studies for immunocompetence

In total, GWAS revealed 477 SNPs associated with 17 immune relevant traits in LR and LW. With the exception of some traits, at least one SNP within most of the genomic regions of interest, identified according to q-value, was also significant after Bonferroni correction. Annotation to the reference genome Sscrofa 11.1 identified 113 genes in LR and 71 genes in LW. Results are partly in accordance with immune-related QTL published in previous studies (e.g., [10, 31, 33–35, 40, 42, 47]). The analyses showed clear breed differences. In LR (n _*SNPs*_ = 280) more significant associated SNPs were found, compared to LW (n _*SNPs*_ = 197). Moreover, all of the markers were breed-specific. This might be due to a strong impact of the breed on baseline values of the immune system [48]. Across-breed differences were probably caused by selection processes and the heterogeneity of the genetic structure within the breeds.

Due to the comprehensive results, the discussion focuses on the importance of the networking structure of immune relevant traits and trade-offs between the immune system of piglets and economically important fertility and production factors.

Analyses on WBC in LR identified significant associations on SSC4, SSC12 and SSC14. The genomic region on SSC14 (41.7 - 48.5 Mb) confirms a QTL for WBC in a German LR population [10] and overlaps with QTL for platelet count and plateletcrit [31]. In addition, a SNP for haemoglobin, haematocrit and RBC in a Duroc × Erhulian population has been revealed close to our result on SSC12 [34].

An intron variant (*MARC0040388*, 34.5 Mb) within the gene *lactoperoxidase* (LPO) on SSC12 showed chromosome-wide significance for neutrophils in LR. Pathway analysis indicates LPO to affect neutrophils in their phagocytolytic activity [49, 50]. A previous study in German LR has revealed a QTL for mean platelet volume in an enclosing region on SSC12 [10].

Our study revealed significant associations for monocytes in both breeds. In LR, the genomic regions for monocytes and neutrophils were located close together (<1 Mb) at 36 Mb to 37 Mb without any intersection. From the physiological point of view, relationships between neutrophils and monocytes are well known, especially in the innate immune system and the defense against pathogens [51]. Use of whole genome sequencing data might clarify the underlying genetic structure. In LW, results on SSC18 were found within QTL associated with mean platelet volume [10] and CD4+CD8- T cells and the ratio of CD4+ to CD8+ T cells [47].

Especially cytokines, as intercellular signalling molecules, are pleiotropic and interconnected in the mechanisms of the immune system [8, 40]. Nevertheless, literature on the genetic background in pigs is limited and underlying mechanisms of interaction in cytokines have not been investigated thoroughly. Therefore, this study reveals mostly novel insights into the genetic background affecting cytokine profiles in pigs under non-challenge conditions. The current study detected 23 genome-wide significant SNPs on SSC15 associated with IL-8 in LR. One of them is an intron variant (*ASGA0070620*, 120.4 Mb) within the gene *transmembrane BAX inhibitor motif containing 1* (TMBIM1). TMBIM1 is involved in the neutrophil degranulation and thereby, for instance, in regulating the exocytosis of inflammatory mediators [49, 50, 52, 53]. In humans, IL-8 is well known to affect neutrophils, for instance through priming or adhesion [52].

GWAS on IL-12 concentration in LW piglets provided a moderate number of immune relevant candidate genes, inter alia *RNA binding motif single stranded interacting protein 3* (RBMS3). Significant markers within RBMS3 were located within a QTL previously described to be associated with IL-10 level [42]. Significant markers for IL-12 in LW seem to be important in immune system processes as has been already shown by previous studies, reporting QTL associated with MCH and MCV in LR [10] and CD4+CD8+ percentage [47].

Furthermore, association analysis on TNF- *α* in LW detected significant SNPs on SSC1 and SSC9. An intron variant on SSC1 (*ASGA0001781*, 26.7 Mb) and an intergenic variant on SSC9 (*ASGA0097568*, 138.5 Mb) exceeded the genome-wide level.

The genetic component of the biological dam in the development of piglets’ immune system still remains unclear. The results indicate consequences on IL-6 in LW piglets depending on the corresponding cytokine status of the biological dam. Nevertheless, most of the genomic regions from basic analysis with model (1) were confirmed after the environmental correction. However, putative pleiotropic mechanisms in cytokines were supported in all statistical models.

Knowledge about pleiotropic mechanisms might help to clarify genetic relationships between the traits [54]. Indications on these putative pleiotropic regions were also observed within the here studied breeds LR and LW. In total, we identified two across-breed regions and three additional regions with putative pleiotropy in LR or LW including all trait complexes investigated in this study.

In LW, association studies identified a chromosome-wide significant SNP (*ALGA0085557*, 56.0 Mb) on SSC15 in haemoglobin and haematocrit. Located on SSC16, a SNP (*ASGA0074790*, 78.0 Mb) was found which was significantly associated with haemoglobin, haematocrit as well as RBC. Therefore, in red blood cell characteristics, pleiotropy in LW is assumed. These potentially pleiotropic SNPs underline the highly interacting mechanisms of the immune system. According to the biological context, this relationship is not unexpected. Yan et al. [35] reported consistently high genetic correlations between haemoglobin, haematocrit and RBC at day 18 (*r*_*g*_ 0.77-0.85) and day 46 (*r*_*g*_ 0.50-0.77).

In LR, a genomic region on SSC17 showed nine identical SNPs significantly associated with at least two of the cytokines IL-1 *β*, IL-4 and IL-10. One of these SNPs is a downstream gene variant of the gene *reactive oxygen species modulator 1* (ROMO1). Among others, ROMO1 is classified in the biological process of a defense to a bacterium [55, 56]. Human monocytes and macrophages have shown an overexpression of the protein Romo1 in case of tumor disease. In this context, an inhibition of the immune response has been assumed [57].

Furthermore, we identified two regions on SSC5 and SSC12 with a size of around 3.6 and 3.9 Mb including three and 16 SNPs, respectively. Markers were associated with up to three different immune traits in LR and LW.

The adjoining SNPs on SSC5 were associated with MCHC in LR and haemoglobin in LW. *Fibroblast growth factor 6* (FGF6), as a putative candidate gene within the described genomic region, is involved in signalling processes and thereby in cell growth and survival processes due to the Phosphatidylinositol 3-kinase cascade [49, 50, 58]. In addition, FGF6 was recently annotated to the current reference genome Sscrofa 11.1 [59]. Another of these markers (*ASGA0025952*, 65.8 Mb) is located within the gene *A-kinase anchoring protein 3* (AKAP3). It is a missense variant probably causing an amino acid change. Biological pathways in humans indicate a link to signalling by regulating the mitogen-activated protein kinase (MAPK) pathway [49, 50].

A genomic region on SSC12 was detected around 50.1 to 54 Mb associated with different cell parameters of the immune system and the blood. SNPs revealed a significance for monocytes and neutrophils in LR and RBC in LW. A putative candidate gene is *CST telomere replication complex component 1* (CTC1), directly associated with the percentage of neutrophils in LR on SSC12. Findings in humans outline CTC1 is involved in immune system processes and the development of the immune system [55, 56, 60].

Conserved functions might have an effect across species [24], and, thus, it is recommended to consider TMBIM1, RBMS3, ROMO1, FGF6, AKAP3 and CTC1 as putative candidate genes, although they are not yet fully described for pigs in detail.

*Genetics of immunocompetence and economic traits*Taking the identified markers into consideration for further analyses, we need to check for undesirable relationships with traits involved in the survivability and diseases susceptibility but also regarding production traits and meat quality.

In this context, the current study identified markers associated with neutrophils in LR (SSC12), IL-8 concentration in LR (SSC15) as well as IL-10 and IL-12 concentration in LW (SSC1 and SSC13). Genomic regions were identified close to QTL for intramuscular fat content [61–63] or within a QTL previously reported for the genetic defect cryptorchidism [64].

Additionally, our findings for IL-8 in LR and MCHC in LW indicate a connection to QTL regions associated with feed conversion ratio and average daily gain in Duroc [65]. The genomic region on SSC14 identified in GWAS for WBC in LR was found close to a QTL previously described for ham weight in a LW population [66].

Results of this study indicate overlaps between regions significantly associated with immune relevant phenotypes and QTL for survivability of piglets and growing pigs. A putative relationship is supported by several studies which were summarized in the review of Heuß et al. [6]. In this regard, important results in our study were markers on SSC13 associated with the concentration of IL-12 in LW and markers associated with neutrophils in LR. These regions have been previously described to be associated with the number of stillborn [67, 68].

Furthermore, significant SNPs on SSC14, associated with WBC in LR and RBC in LW, have been previously described in the context of the reproductive traits litter size (WBC) [67] and mummified piglets (WBC, RBC) [68].

The relationship between the immune system and the survivability of piglets gains in importance. Besides, several putative pleiotropic regions have been discussed in “Genome-wide association studies for immunocompetence” section. In this context, the identified across-breed genomic region on SSC5 was additionally described to be associated with litter size in Duroc [69].

## Conclusions

In summary, the study identified 477 SNP markers associated with immune relevant traits. Analyses were performed in piglets of a LR and LW population kept under high hygienic standards and without challenging the immune system. GWAS results pointed out clear differences between the breeds LR and LW. Comparison between previous studies and findings in this study indicate a complex genetic background of immune relevant traits and health indicators. Several interesting genomic regions suggest a pleiotropic background. Further investigations need to follow, considering relationships and antagonistic mechanisms between the immune system, survivability, performance traits and other economically important traits. In general, as health and immune traits are expected to become an essential part of balanced pig breeding, this study provides first insights into regions with special importance for the immune system of piglets.

## Methods

### Animals, sample collection, phenotyping

A total of 535 piglets (♂190/♀345) of LR and 461 piglets (♂170/♀291) of LW were analysed. Animals were a subset of the nucleus populations which aim to reflect the genetic variability of both populations with respect to their different breeding objectives. All piglets were born between 2015 and 2017 on five farms of the German breeding organization BHZP GmbH (Bundeshybridzuchtprogramm GmbH). All herds were subjected to high hygienic standards. The experimental design aimed to sample a triplet consisting of the mother and two full siblings born alive.

Piglets were routinely blood sampled in the week around weaning (⌀ 42 to 44 days), sows around piglets’ birth. Sampling was performed by collecting blood via the *Vena jugularis*.

Traits investigated are listed in Table 5. Traits of the complete and differential blood count were determined using the optoelectronic technology ADVIA^®^ 2120. Band cells as well as basophils and eosinophils were excluded from further analyses due to low expression, resulting in a variation close to zero. Haptoglobin concentration was measured using a photometric technology. Cytokines were analysed with Millipore’s *Milliplex Map Kit Porcine Cytokine/Chemokine Magnetic Bead Panel* on a Luminex^®^ 200, as well as the software xPonent 3.1. *Milliplex Map Kit* enabled simultaneous analysis of eight cytokines. Cytokine concentrations below the analytical threshold were replaced by one-half of the threshold [70, 71]. Measurements which did not fulfill the quality criteria determined by the laboratory technique were excluded from further analyses. Cytokines and haptoglobin measurements were log-transformed to assume a normal distribution and to take the skewness of distribution into account.

**Table 5:** Immune relevant traits investigated in piglets from a Landrace and Large White population

Complete blood count	Differential blood count [*%*]	Cytokines [ng/mL ]
White blood cells [G/l ]	Lymphocytes	Interferon- *γ* (IFN- *γ*)
Red blood cells [T/l ]	Monocytes	Interleukin-10 (IL-10)
Haemoglobin [g/l ]	Band cells	Interleukin-12 (IL-12)
Haematocrit [l/l ]	Neutrophils	Interleukin-1 *β* (IL-1 *β*)
Mean corpuscular volume (MCV) [*fl*]	Eosinophils	Interleukin-4 (IL-4)
Mean corpuscular haemoglobin (MCH) [*pg*]	Basophils	Interleukin-6 (IL-6)
Mean corpuscular haemoglobin concentration (MCHC) [g/dl ]	Other cells	Interleukin-8 (IL-8)
Platelets [G/l ]		Tumor necrosis factor- *α* (TNF- *α*)
Haptoglobin [mg/ml ]		

Descriptive statistics including least-squares means and breed differences are listed in Table 1. Model (0) was used to test for differences between the breeds. Correction was performed for the fixed environmental factors of breed (*ΔBR*), age (*ΔA*) and weight (*ΔW*) at sample collection and their interaction, a combined herd, year, season, sex effect (*HYSS*) and litter number (*L*). The intercept is represented by *μ* and regression coefficients are given as *b*_1_,*b*_2_,*b*_3_ and *b*_4_ and environmental residual effects as *e*. In the analyses of cytokines, batch (*B*) was an additional fixed effect and biological dam (u _*d*_) an additional random effect.

Model (0):

$${}\begin{aligned} y_{{ij}} &= \mu + (b_{1} * \Delta BR) + (b_{2} * \Delta A) + (b_{3} * \Delta W) \\ &\quad+ (b_{4} * \Delta A \Delta W) + HYSS_{i} + L_{j} \ [+ B_{k} + u_{d}] + e_{{ij}} \end{aligned} $$

Note: Terms in [ ] are only used in the analysis of cytokines.

### Genotyping

Blood and tissue samples were analysed using the PorcineSNP60v2 BeadChip (Illumina, San Diego, CA, USA).

Breed-specific quality control on autosomes was applied using the R-package GenABEL [72]. Samples and markers with a call rate <95% and markers with a low minor allele frequency (<1%) or high linkage disequilibrium (*r*^2^>0.8) in a region of 3 kb were excluded from further analysis, resulting in 41’872 (LR) and 42’388 (LW) markers in 534 (LR) and 461 (LW) piglets.

Genetic distances were visualised using multidimensional scaling based on a genomic relationship matrix calculated within the R-package GenABEL [72] (Fig. 1). Consequently, the two populations were analysed separately.

### Statistical analyses and genome-wide association studies

In a first step, data were analysed with a generalized linear mixed model (model 1). Observations (y) were corrected for the fixed environmental effects age (*ΔA*) and weight (*ΔW*) at sample collection and their interaction, a fixed SNP effect, herd, year, season and sex, combined to a HYSS-effect (9 levels in LR, 13 levels in LW), and litter number (*L*, 1 - >4). The intercept is represented by *μ* and regression coefficients are given as *b*_1_,*b*_2_,*b*_3_ and *b*_4_. The environmental residual effects were denoted by *e*. In the analyses of cytokines, a fixed batch effect (*B*, 20 levels in LR, 19 levels in LW) and the random effect “biological dam of the piglet” (u _*d*_) were added to the model. Both additional effects have been consistently identified as relevant for cytokines.

Model (1):

$${}\begin{aligned} y_{{ij}} &= \mu + (b_{1} * \Delta A) + (b_{2} * \Delta W) + (b_{3} * \Delta A \Delta W) \\ &\quad+ (b_{4} * SNP) + HYSS_{i} + L_{j} \ [+ B_{k} + u_{d}] + e_{{ij}} \end{aligned} $$

Note: Terms in [ ] are only used in the analysis of cytokines.

In a second step, the impact of direct environmental maternal effects on the piglet’s immunocompetence were verified using model (1^*^). Accordingly, model (1) was extended by the corresponding blood parameter of the biological dam, modelled as a fixed covariate. Phenotypic information of all biological dams in LR (n = 262) and LW (n = 226) was available. In case of a significant effect of the covariate, the trait was considered for GWAS using model (1^*^).

Covariables age, weight and blood parameter of the biological dam, used in models (1) and (1^*^), were standardised to a mean value of zero.

The R-package GenABEL [72] with a generalized linear model approach was used to analyse all parameters except for the cytokines. In order to identify associations between SNP genotypes and phenotypic information, a fast score test was conducted. Because of limitations in the GenABEL-package, the analysis of cytokines was performed using ASReml^®^ [73], which is more flexible in the inclusion of random effects. SNP effects were estimated one by one. *P*-values were assessed based on the effect and its standard error, assuming a normal distribution.

Lambda (*λ*) values were low to moderate in LR (0.99-1.87) and LW (0.95-1.67). Adjustment for biases through the population structure was performed according to the Genomic Control approach [74]. After correction, the *λ* values were in an acceptable range <1.08. In addition, correction for a modified false discovery rate [75, 76] of 5% was applied in order to take multiple testing into account using the R-package qvalue [76]. Q-values <5% were denoted as genome-wide/ chromosome-wide significant, based on the number of tests (total number of SNPs/ number of SNPs per chromosome). To test for a more stringent threshold in terms of multiple testing, additional validation through Bonferroni correction was performed using *p.adjust* R-command. Markers with adjusted *p*-values <0.05 and 0.1 were highlighted in Additional files 1 and 2.

The phenotypic variance explained by a single SNP was calculated according to the transformation of a student’s t-distribution into a z-distribution [77] using the following formula:

$$Var\ [\%] = \frac{\chi^{2}_{1df}}{N - 2 + \chi^{2}_{1df}} * 100 $$

### Annotation of significant SNPs

Markers were mapped to the porcine reference genome Sscrofa 11.1 and variants were identified according to Ensembl release 100 [78]. In addition, significant markers were examined regarding their biological function and position in putative candidate genes. Classification into known pathways was implemented using Reactome [49, 50]. Furthermore, a comparison with the literature deposited in the AnimalQTL database [25] was conducted.

## Supplementary Information

**Additional file 1** Complete GWAS results in Landrace. Significant SNPs in Landrace associated with immune relevant traits identified by GWAS. MCHC: Mean corpuscular haemoglobin concentration, IL: Interleukin, SSC: Sus Scrofa Chromosome, MAF: Minor allele frequency, *: genome-wide significance, adj. *p*-value: adjusted *p*-value after Bonferroni correction, B5: Additionally significant after Bonferroni correction with adjusted *p*-value <0.05, B10: Additionally significant after Bonferroni correction with adjusted *p*-value <0.1.

**Additional file 2** Complete GWAS results in Large White. Significant SNPs in Large White associated with immune relevant traits identified by GWAS. MCHC: Mean corpuscular haemoglobin concentration, IFN: Interferon, IL: Interleukin, TNF: Tumor necrosis factor, SSC: Sus Scrofa Chromosome, MAF: Minor allele frequency, *: genome-wide significance, adj. *p*-value: adjusted *p*-value after Bonferroni correction, B5: Additionally significant after Bonferroni correction with adjusted *p*-value <0.05, B10: Additionally significant after Bonferroni correction with adjusted *p*-value <0.1.

## Data Availability

Data cannot be made publicly available, as they are owned by a third party, the BHZP GmbH. The datasets used and analysed during the current study are available from the corresponding author on reasonable request and with permission of the BHZP GmbH pig breeding company (henne@bhzp.de).
